# How Do Paediatricians Manage Comfort with Uncertainty in Clinical Decision-Making

**DOI:** 10.5334/pme.1394

**Published:** 2024-10-22

**Authors:** Colin J. McMahon, Muirne Spooner, Matthew Sibbald, Maryam Asoodar

**Affiliations:** 1 Department of Paediatric Cardiology, Children’s Health Ireland at Crumlin, Dublin, Ireland; 2 School of Medicine, University College Dublin, Belfield, Dublin 4, Ireland; 3 School of Health Professions Education (SHE), Maastricht University, Maastricht, Netherlands; 4 Department of Medicine, Royal College of Surgeons University of Medicine and Health Sciences, Ireland; 5 Department of Medicine, Faculty of Health Sciences, McMaster University, Hamilton, Ontario, Canada

## Abstract

**Background:**

While healthcare practice is inherently characterised by uncertainty, there is a paucity of formal curricular training to support comfort with uncertainty (CWU) in postgraduate training. Indeed, some evidence suggests medical training inherently conflicts with CWU in emphasizing pedagogies focussing on “fixing” the problem. While referral patterns increase significantly, dealing with uncertainty has direct implications for patient referral rates and use of valuable healthcare resources.

**Methods:**

Paediatricians in Ireland were invited to participate. Face-to-face interviews were conducted after participants watched videos of varied clinician-patient interactions.. Two researchers independently analysed the collected data using thematic analysis. Triangulation and member checking was performed to ensure validity of findings. A reflection journal documented the research journey.

**Results:**

Thirty four paediatricians participated. Five themes were identified: the interplay between quality of information, uncertainty and decision-making, confidence in clinical assessment and first-hand patient evaluation, anxiety and fear experienced by medical professionals when dealing with complex and serious conditions, strategies employed by medical professionals in managing their own uncertainty and the impact of societal and parental expectations on medical decision-making. These are moderated by a number of factors, most significantly the child’s caregivers’ comfort with doctors reassurance (CDR). Enacted management will diverge from the consultant’s clinical plan when the caregiver’s CDR cannot be satisfactorily supported.

**Discussion:**

Clinician CWU in the paediatric context is inextricably linked to caregiver CDR. The complexities and central importance of social context in understanding CWU has important implications for how we develop educational activities to support clinician CWU and patient/care-giver CDR. This may translate to efficient use of limited resources in healthcare settings.

## Introduction

Decision-making in medicine is often challenging and frequently confounded by some degree of uncertainty [1]. Whether comfort with uncertainty describes a trait (a personality characteristic) or a state (a modifiable way of experiencing a phenomenon) has much debated. There is a narrative around learners’ experience of uncertainty, which has often been negative [2] and evidence of reluctance to engage in management of uncertainty [3]. This is perhaps due to conceptualisation of it as a trait, that is, a stable personality characteristic, which cannot be changed [4]. There is increasing value on preparing clinicians to manage their tolerance of ill-defined problems in clinical practice [5,6,7,8,9,10,11].

In health professions education, there is a move to consider comfort with uncertainty (CWU) as a lived experience that, therefore, is amenable to modification via training [5]. There are a number of reasons to support this view. How learners approach uncertainty tends to vary widely [12,13] However, a common “maturation” process by which many clinicians report gaining CWU as they acquire more experience, is described by Moffett et al [2]. Additionally, the same individual may show different responses to uncertainty in discrete situations [14]. How this happens and manifests in the senior clinician requires more exploration. It is recognised that responses to uncertainty include cognitive, emotional and behavioural aspects [15]. Clinicians’ comfort with uncertainty occurs through a number of metacognitive activities, which are shaped by perceptual, emotional, and situational cues [5].

Ilgen identifies that clinicians employ a number of activities in managing uncertainty, which include “borrowing” and “handing over” certainty, which can be exemplified by informal discussion with colleagues or formal referral, respectively [16]. There is a suggestion that discomfort can increase investigation orders [17,18] and referral rates [19,20]. The resource implications of these actions include increasing referral rates for benign conditions, lengthening waiting lists and increased patient anxiety [21].

Patient comfort with uncertainty impacts their outcomes. When clinicians perceive patients to have poor comfort with uncertainty, they are more likely to withhold information and less likely to recommend interventions with uncertain outcomes [22]. Patients with high comfort with uncertainty consistently demonstrate better emotional well-being [15]. These studies highlight the value of clinicians learning how to manage their own uncertainty and that of their patients and caregivers.

Much of the literature is interested in curricular development to support junior clinicians in managing uncertainty [3,23]. Previous work has provided insight into how senior clinicians manage uncertainty in academic emergency departments [16]. Given the dramatic increase in referral patterns to paediatric clinics, the capacity to deal with uncertainty may have real tangible effects on waiting lists and how limited resources are allocated [21]. Our work is novel in exploring senior paediatricians’ responses to uncertainty working across diverse contexts (i.e. tertiary referral urban centres, general hospitals, rural practices).

## Definitions

**Figure 1:**
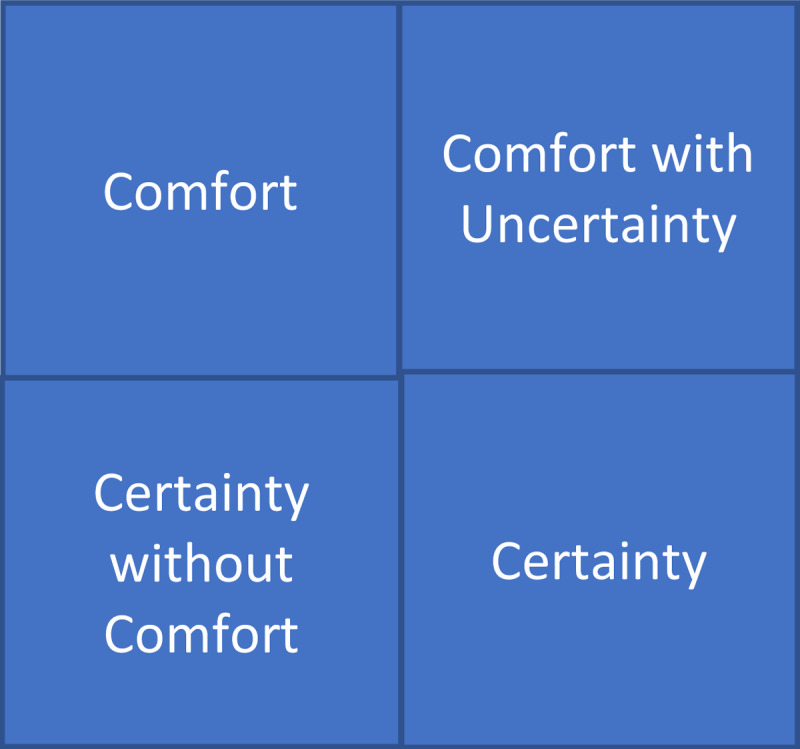
Comfort With Uncertainty (Ilgen model [5]). The key elements in dealing with uncertainty.

- Ilgen et al. define four components to ‘comfort with uncertainty’ (Figure 1) [5]. Comfort is confidence in one’s ability to act, while certainty is confidence in one’s own interpretation of a clinical scenario. Uncertainty is defined as conscious, metacognitive awareness of ignorance. Comfort with uncertainty is therefore the ‘phenomenological lived experience of having the confidence to act on a situation without the full confidence of completely understanding the underlying mechanism or reasons for the problem’. Certainty without comfort describes a condition where one knows exactly the mechanism or reason for the problem but has no idea how to manage it. Uncertainty may envelop the diagnosis of a condition, the treatment strategy or options for a problem, or possibly both the diagnosis and the treatment options.

## Theoretical framework

Comfort with uncertainty (CWU) is highly contextualised by individual, environmental and relational aspects [2]. Several taxonomies of uncertainty have been proposed to inform a shared concept of uncertainty [24,25]. Given its particular relevance to health care professionals, we use Ilgen’s [5] theoretical framework and definitions of certainty and comfort with uncertainty (Figure 1).

The research questions in this study are:

1. How does uncertainty in clinical cases impact the decision-making process of pediatricians, and
2. What strategies do they employ to manage this uncertainty?

## Methods

### Setting and study design

Paediatricians based in Ireland were eligible to participate in this study. The study involved viewing a simulated clinical scenario, which depicted a young child presenting symptoms of a murmur. We chose this scenario as it commonly presents to the outpatient paediatric and cardiology clinic, demonstrating a young child with an asymptomatic murmur in which the clinician completes a thorough history and physical examination. This aligned with the CWU theoretical framework as the potential management was left uncertain and dependent on the clinician’s level of CWU. The video materials were reviewed by two independent paediatric cardiologists who confirmed it aligned with a typical clinical presentation.

### Participants

Participants from urban and rural centres with differing levels of service and experience were recruited by email invitation from first author (CM) to enroll in the study. All participants were paediatricians practising as general paediatricians or paediatric specialists.

### Data collection

After the participant paediatrician watched the video of the clinical encounter between the general paediatrician, the child, and the mother (clinical scenario outlined in the semi-structured interview scheme), they underwent a semi-structured interview, which was transcribed. The first author (CM) conducted all the interviews. These interviews took place at the participants’ respective hospitals, requiring CM to travel across the country. CM personally drove to each hospital to conduct the interviews. We do not believe that the interviewer’s background influenced the interview guide or the analysis. The purpose of the interview was to explore the participants’ lived experiences with similar problems, whether they detected any red flags or cues (reassuring or concerning) from the video, their proposed course of action, the factors that build their comfort with uncertainty, and how they manage this uncertainty for caregivers and patients in similar situations.

### Clinical Scenario and Semi-structured Interview

The participants were initially introduced to a brief scenario (outlined in the semi-structured interview scheme), followed by a semi-structured interview. See the Appendix for the complete interview guide, which contained a range of questions including, but not limited to: Question A and Question B (below).

*Kate is a five-year old asymptomatic girl who presents to her general paediatrician with a vibratory grade 2/6 cardiac murmur which reduces on sitting up and Valsalva manoeuvre. There are no red flags. The paediatrician reassures the mother this is an innocent murmur. The mother remains worried however and demands further cardiac investigations. The paediatrician tries to reassure her uncertainty but she insists on an echocardiogram. They are referred to cardiology and will wait for 2 years for a routine outpatient appointment. Kate and her mother are now convinced she has a heart problem because why would the doctor refer to the cardiologist unless there was a problem*.

Some examples of questions posed to the participants included:

***A.** What did you make of the clinical interaction between doctor, child and parent?*
***B.** Were there any red flags you witnessed from the clinical interaction?*

### Analysis

To ensure a comprehensive understanding, a triangulation of sources was employed. This involved the use of data from paediatricians with different levels of expertise. The transcribed interview data (otter.ai) underwent an inductive thematic analysis independently by two of the investigators (CMM and MS) to allow for the identification, analysis, and reporting of patterns within the data. Both investigators identified common themes collaboratively, bringing distinct perspectives from their respective branches of medicine. This approach drew on their varied backgrounds and experiences to enhance the analysis and ensure a more comprehensive exploration of the data. A reflection journal was maintained to document the research journey, providing a personal account of the decisions made, the issues encountered, and the lessons learned. In addition to the researcher’s journal, to further enhance the credibility of the findings, member checking was performed.

## Results

Thirty-four paediatricians (20 female; 14 male) participated (Table 1). The median time practicing as a consultant paediatrician was 12 years (range 4–35 years). Twenty-two doctors worked in a large city/urban hospital and twelve worked in a regional/rural hospital (C and R respectively). The C and R codes are used below to reference where the participants are working. Then the years of experience and the field of expertise are mentioned in the referencing of the quotes, e.g. (R28: 26 years; Paediatrics)

**Table 1:** Demographics of Study Participants.


Total Number Participants	34

Female	20

General Paediatricians	23

Subspecialists	11

Emergency department paediatricians	4

Respiratory paediatricians	3

Infectious disease	1

Immunology	1

Metabolic disease	1

Palliative care medicine	1

City Hospital	22

Rural or Regional Hospital	12


Paediatricians describe varying levels of comfort with uncertainty in relation to clinical cases as stimuli. Positive reactions to uncertainty were more commonplace in all areas. We identified five themes, which we describe below.

### The Interplay Between Quality of Information, Uncertainty, and Decision-making

Participants were confident in making a diagnosis and management plan for the video scenario presented, mainly as there was minimal epistemic uncertainty (i.e. they had access to a clear history and presentation of comprehensive clinical findings). A senior paediatrician emphasised that this confidence stems from their ability to critically appraise a situation when they have good quality information:

*Number one is what’s the quality of the information I have. If I have good quality information, I can make a good decision. the first step is to collect and gather, better information. (R28: 26 years; Paediatrics)*

Furthermore participants mentioned that they experience more doubt with cases where the history is vague, which leads to differing actions for the same case:

*I’m working with some doctors I have seen when they don’t feel confident with the quality, they are not looking for more, a bigger amount of information but they try to replace the quality by ordering more tests. So if you don’t have a good history of the murmur, you’ll do the tests (R28: 26 years; Paediatrics)*

Several paediatricians of varying experience discussed the presence of “red flags” as triggering uncertainty, and described the internal checklist they considered:

*…well child started preschool, keeping up with their peers, there’s no symptoms of cardiac failure or anything, able to exercise, never breathless, no cyanosis. And so all that was good, and it was an incidental murmur that was picked up because the doctor was just reviewing anyway. And then in terms of the examination then, there was no signs except for a grade two to three murmur that was non-radiating to left sternal edge. . So the pulses were good saturations were normal. No difference between pre and post saturations so all those are fitting with its most likely an innocent murmur. (R25: 5 years; Paediatrics)*

A broad range of paediatricians mentioned their appropriate discomfort with uncertainty in the setting of pathological findings or red flags:

*the most important thing is, is the growth of the child’s whether there’s any associated symptoms, such as breathlessness, difficulty feeding diaphragm, perspiration with feeding, particularly if the pericardium is quite active. I think that’s an important sign. And if the second heart sound is very loud, if the murmur the nature of the murmur is harsh, if there, if it radiates rather than being localized, and if it doesn’t vary with posture that would be important finding, and then also the pulses if the pulses are significantly reduced in the lower or upper limbs. That’s always a concern to me. (U13: 30 years: Paediatrics)*

A reassuring finding repeatedly described by an experienced paediatricians in the emergency department was if:

*the murmur disappeared when she sat up, that she was pink, that she had no radio femoral delay, that her femoral pulse volume was good, that her vitals are normal. (U14: 9 years: Emergency room)*

### Confidence in Clinical Assessment and First-Hand Patient Evaluation

Participants explained that access to a first-hand assessment of the patient is the main moderator of CWU here. They attribute high value to their clinical skills and harness their confidence in clinical assessment to mitigate uncertainty:

*She is a well, thriving child who has essentially normal observations, normal saturations, normal respiratory rate, no hepatosplenomegaly, radio femoral delay and the murmur changes with position so there is no concerns. (U18: 6 years; Paediatrics)*

*I suppose your clinical skills sort of reassure you that mostly murmurs tend to be innocent (U19: 13 years: Respiratory)*

Conversely, their confidence shifted towards feeling doubt when they were unable to complete a history and physical exam. This could be due to stimulus characteristics, e.g. a distressed child who may not cooperate with assessment. It was a situational characteristic during the pandemic, when remote assessments were required, and which notably dissipated as they felt more adept with modifying their approach for tele-assessment:

*When we started it (doing remote consultations), I wasn’t sure, I wasn’t happy with what we are doing, how can we review these patients over the phone when I can’t actually see them in person and cannot do the physical exam? But it came over time, you know. So we have developed those skills, how to assess a child over the phone. (R31: 7 years; Paediatrics)*

They also pointed to the need to be thorough in history and physical examination assessment so that they could adequately assess parental concerns:

*If you don’t take a comprehensive history or examination then you’re not really going to get into the detail about why the parent is anxious about something. (U19: 13 years; Respiratory)*

### Anxiety and Fear When Dealing with Complex and Serious Conditions

An inability to elicit the history or directly examine the patient (e.g. remote consultations by video-link) contributed to anxiety participants experienced. The specific condition or specialty also impacted the degree of anxiety or fear experienced:

*CHD (congenital heart disease) is feared on call. Because the children are very sick, they often have very complex anatomy that people don’t understand. So therefore, their default is to be shying away from it……. I think the natural response, certainly on call, if you haven’t done cardiology and get called about it, is “oh God, am I going to know about this? (U20: 4 years; Paediatrics)*

Participants explained that the “worst case scenario” of each presentation affected their comfort with uncertainty. Several mentioned that cardiac murmurs being associated with Sudden Cardiac Death Syndrome automatically elevates discomfort compared with a clinical condition that is usually benign, e.g. sprained ankle:

*I suppose the heart and the lungs may generate an extra level of anxiety, because you know, you hear people’s heart stopping and reports in the media, or they might have heard of some child on a football pitch, you know, suddenly dropping dead. (U19: 13 years; Respiratory)*

Participants indicated that once they recognised they were having a negative emotional reaction, it prompted further actions, e.g. testing, re-examining, contacting a colleague for advice:

*If I think it’s quite sinister or serious, I may even pick up the phone and talk to someone and see what they think. It depends on the degree of worry. Obviously I’m talking about not necessarily a red flag symptoms but something that’s slightly out of kilter, that just doesn’t sit well with me. And they’re not that doesn’t happen that often, but it does happen. And usually when I’m stuck. I ask for help. (U24: 9 years; Emergency medicine)*

Sometimes the case did not incite anxiety but the potential parent/care-giver reaction spurred fear.

### Strategies Employed to Manage Uncertainty

Participants’ responses to scenarios where they encountered uncertainty could be divided into two main themes, taking action and decision-making, both related to the clinical management plan and taking action and decision-making in relation to managing caregiver comfort with uncertainty.

Participants describe contacting a colleague for advice as a common strategy in responding to and managing caregiver uncertainty:

*interacting with colleagues can be helpful to get their advice on things. (U16: 14 years; Immunology)*

Participants indicate that their personal professional training may affect their CWU:

*Your level of comfort so if you’ve done cardiology, you know, unless you’re a consultant and but you’ve trained in cardiology, no matter how brief whether it’s three months, six months, a year, then your own personal level of comfort or what you feel you can deal with safely is obviously higher. (U5: 18 years: Paediatrics)*

Participants reported infrequent emotional responses to uncertainty. When they did, there were several topics to note. Negative emotions predominated, participants indicating they were strong but intangible reactions:

*You need to embrace the concern, and actually, but you need to embrace the concern, and that’s just sitting inside me and it’s whatever you call it, it’s tension sitting in your abdomen, some people it’s tension in their chest, whatever. (U24: 9 years: Emergency room)*

*Often it’s hard to pin it down, you know. And I think sometimes you have this feeling for a few days before you actually force yourself to sit down and force yourself to kind of go through it and say well, “why am I so worried about this case?” or” what is it specifically about this situation that is making me so anxious? (U16: 14 years; Immunology)*

### Impact of Societal and Parental Expectations on Medical Decision-making

With the specific case watched, almost all participants indicated their clinical plan would be to communicate to the caregivers that the child had an innocent murmur and there was no need for any follow-up. The majority also underlined that this action would not always occur in practice, due to insurmountable caregiver anxiety.

They described parents and societal expectations as being consumerist in terms of patient care. While they were able to tolerate the uncertainty of a case based on a clinical assessment, their actions may not reflect this, due to these external pressures:

*That’s the way people are, they want instant sorting. They don’t want hassles. They don’t want children who have issues. That’s just society now. (R22: 35 years; Paediatrician)*

The participants suggested that the caregiver’s comfort and anxiety influences the medical decision-making. Participants stated that they are conscious of managing moving caregivers from a state of anxiety to calm:

*I think that’s where our job is, to recognise that parents are going to be anxious because their kids are sick, and it’s about reassuring them. (U19:13 years; Respiratory)*

This meant decision-making and actions were affected not just by what they felt was clinically appropriate, but by the caregivers’ comfort with doctors reassurance (CDR):

Interviewer: *So when you tell a mom going through the history and the physical as thoroughly as you have the reasons why you’re comfortable, it’s innocent, that there are none of these red flags. Is it the parental lack of comfort that’s driving the need for referral?*

Participant: *The short answer to that is it is the parent request or concern. So if there is a parent who will take my first recommendation on board that you go home and I’ll write to your GP, I’ll see you again in a year’s time. If you have any issues, or red flags, come back to me. If a parent accepts that on face value with me, yeah, you know, a clinic is a finite amount of time, I would feel fine. But if I make a referral for these children; number one, I make it because there is parental anxiety. (R28: 26 years; Paediatrician)*

## Actions to Manage Caregiver Comfort with Doctors’ Reassurance

In addition to the above themes, we observed insights into the strategies healthcare providers utilized to alleviate the uncertainty in clinical decision-making often faced by caregivers. These strategies, which are crucial for educational purposes, include the establishment of strong relationships, effective communication, active listening, and honesty. The participants also highlighted the role of observational walk-throughs and an open-ended approach in enhancing caregiver comfort. They indicated that these strategies not only improve the consultation experience but also provide ongoing support, ultimately strengthening the caregiver-provider relationship.

In Table 2, we will delve into these strategies, providing participant quotes, to further understand their implications in strengthening the caregiver-provider relationship and enhancing the caregiver’s experience.

**Table 2:** Strategies Utilized To Manage Caregiver Comfort With Doctors Reassurance.


STRATEGY	SUPPORTING QUOTE

**Relationship Building** A strong relationship is a key action in managing caregiver uncertainty that allows healthcare providers to take appropriate actions while ensuring caregivers are comfortable	*It’s really important that and it builds that relationship. Even though it’s a 15-to-20 minute consultation, it builds that relationship, it builds confidence, they feel heard*. (U14: 9 years; Emergency medicine)

**Effective Communication** Participants referred to observation, active listening, and careful language choice in addition to educating caregivers about red flags and reassuring findings	*I feel parental anxiety and the way I interact in my clinic it’s all about communication…. I think you can reassure them if you can effectively communicate what the problem is*. (U19: 13 years; Respiratory)
Participants felt observation of their history-taking and examination as important in building confidence in their assessment:	*they’re very impressed that you’re so thorough with the history and physical exam and communicate it* (U17: 4 years; Paediatrics)
They value active listening in facilitating care-givers to voice their concerns:	*It’s not just listening, its active listening, …. and that makes them feel reassured that you’re listening to their concerns* (U5: 18 years; Paediatrics)
They carefully choose language, assess patient and care-giver understanding, make changes to how they speak as needed:	*It’s quite obvious the words you’re using or the language you’re using or the decision you’re making has got them perturbed* (U24: 9 years; Emergency medicine)
They educate patients- informing of red flags, pointing out reassuring findings, directing to patient information resources:	*you find that the parents get a lot out of the patient information leaflets (R23: 19 years; Paediatrics)*

**Empathetic Communication** The act of attentive engagement and demonstrating you have heard what they said:	*I think, if, if, if the concerns are heard and they’re listened to properly and you communicate everything back to them, I would, in my opinion, I think, 95% of them, or above would accept that, and, and are reasonable and don’t have any, any issues. And I think, not only does it make the consultation better, I think the child’s quality of life potentially is better the quality of life of the parents, the anxiety of the parents is better that they understand it, they’re not worried”*. (U18: 6 years: Paediatrics)

**Walking Them Through the Observed Findings** Logically walking the caregiver through the relevant findings in the history and physical examination was highlighted as important:	*I think this is one of the most important things to be honest, communication, communication, communication is very important. And then you have to be confident yourself you have to show to the parents look, child is asymptomatic exam is perfect and soft murmurs are common in children. So I think yeah, communication very important to the parents*. (U14: 9 years; Emergency medicine)

**Combining Communication Skills Strategies** Specific combinations of strategies include introducing yourself, sitting at the same level, establishing eye contact, taking your time not rushing the consultation and being thorough:	*Well, it begins at the start to the consultation and it is walking into the room introducing yourself establishing a good rapport with child and parent and essentially, going through as you should do your structure for this scenario in a very thorough fashion. And the family need to feel that that is done in a thorough fashion. So cannot be rushed or felt to be rushed. I will even how I sit with them as opposed to stand in the room. There’s a psychological importance to that…… if you sit with the family, they perceive the clinical interaction as being much longer than if you stand and your eye-line is at least at level or below the person and not above and domineering and, and being honest with you then the language you use is tailored to their, you know, level of education and literacy with respect to medical terms…. I will often say the same thing, a couple of different ways, if I sense that there is a lack of understanding. And I will often ask the family to repeat back to me, what their understanding of what’s happened*, (U24: 9 years; Emergency medicine)

**Honesty** Participants mentioned that the relationship between the provider and the patient or caregiver is of paramount importance. This relationship is built on honesty. Participants indicated that honesty fosters trust, a crucial element in any relationship, but particularly so in healthcare where decisions can have significant consequences. A healthcare provider with 18 years of experience in Paediatrics shares their perspective on this matter:	*Being honest is really important for trust. I think there needs to be trust between us. They need to feel that you’re being straight with them. And I think if they feel you’re hiding something from them or that you know something they don’t, that wrecks the relationship because if you’re trying to say to them, we’re happy if they trust you, then they’ll take that information on board, and they’ll say, Okay, if you’re happy and we trust you, then that’s fine. Whereas if you don’t have their trust, then things may linger on and then when other decisions come up, not in this context, but in any context, then you’re at nothing, because they need to trust you when difficult decisions might come up then*. (U5: 18 years; Paediatrics)

**Leaving the Door Open** Participants were very transparent with caregivers highlighting that there can be situations where the child’s status may change. Rather than exacerbating the caregivers anxiety, this strategy aimed to leave the door open and reassure the parent that if there was any change or deterioration in their child, they could immediately return for further assessment. This safety net provided the caregivers with significant reassurance and helped quell discomfort with uncertainty they may have experienced. Some participants referenced this as a recurring strategy to reassure parents, sometimes even telephoning parents to check on their child:	*I think for me in emergency medicine for instance the febrile child is a scenario which I will be hugely familiar, with head injuries and other things. And we have terminology called safety netting and safety netting is being very open with the families and saying, “I have examined your child, I understood what’s happened, and this is what’s going on from my perspective, this is the diagnosis, however, I appreciate that I am viewing your child at a point in time. And should any of the following things happen, this gives you absolute licence to return to me, and we’ll take it from there essentially”, always saying that I do not expect that to happen, but it’s better that you know about this, than not know about us. “And, and I use that in a huge amount of scenarios…and it’s, I’ve never known it not to work for me. (U24: 9 years; Emergency medicine)* *There is nothing in it to be concerned about. But leaving the door open that if there are things, explaining to her that if something changes that clearly you’re more than happy to see her again. (U5: 18 years; Paediatrics)* *If the parent is extremely anxious, I would give regular phone calls so then knowing when their next appointment is when I go to re-evaluate is really helpful. And having the same person see again so not having them see the registrar or SHO, they come back to me, and then knowing that they can contact us between appointments, and not waiting for them to ring with a symptom, giving them a kind of a regular follow up so they know you’re going to have a follow up so that they’re not getting anxious about what’s going to happen*. (R21: 20 years; Respiratory)


## Discussion

**Figure 2:**
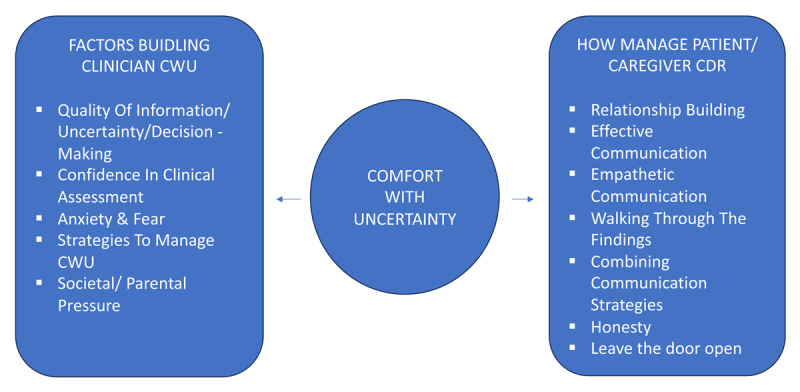
Key components underlying doctor comfort with uncertainty (CWU), interaction with patient/caregiver comfort with doctor reassurance (CDR) and strategies to manage patient/caregiver CDR.

This study is novel in exploring the experiences with uncertainty of a wide range of paediatricians at diverse sites. We sought to explore how paediatricians manage their own uncertainty and also what strategies they employed to help manage caregiver or parental discomfort with uncertainty. The five themes we identified show that pediatricians describe varying levels of comfort with uncertainty in relation to clinical cases as stimuli. These themes are inter-related (Figure 2) and the fifth theme is intricately related to potential modifying actions medical professionals can use to manage caregiver uncertainty.

Uncertainty is universal, in life as well as in the clinical environment [26]. Uncertainty in decision-making for even simple cardiac lesions [27] as well as learning new technical skills [28] has been well recognized. Decision-making is further complicated by inherent biases [29] and also challenges in consistent decision-making [30]. Our participants highlighted the interactive challenge in dealing with uncertainty and decision-making and the importance of having accurate information. This was particularly pertinent to our study as it was conducted during the COVID-19 pandemic, a period that embodied uncertainty [31]. The importance of a really accurate history and physical examination was repeatedly mentioned as building CWU [32]. The primacy of the history and physical examination, if neglected, had serious consequences, with clinicians opting to pursue testing as a surrogate. Conversely, vague symptoms or lack of clarity in the history or the physical findings damaged clinician CWU. Clinicians rely on red flags and cues to alert them to their being a problem [16]. Their position on the spectrum of CWU may vary depending on these red flags and cues.

Perhaps the most important factor in guaranteeing quality information gathering was the first-hand interaction of the doctor and the patient (care-giver). This afforded the paediatrician reassurance and confidence that their history and physical findings were accurate. It also enabled them to checklist red flags and rule out worrying cues that would support them in building CWU. Some professionals mentioned the importance of “gut feelings” and use of heuristics to alert them to there being a real problem [33–34]. Some authors have advocated for greater use of smart heuristics in a world that is increasingly volatile, uncertain, complex and ambiguous (VUCA), reinforcing the importance of employing ‘gut feelings’ in activating awareness of pathology or ‘something just not right’ [35].

Comfort with uncertainty is not a fixed entity, but is rather context dependent varying with different patients, dealing with different medical problems [36]. Complex or high-stakes medicine such as paediatric cardiology or emergency room settings may activate greater feelings of anxiety or discomfort with uncertainty as highlighted by some paediatricians in this study [37]. This may arise due to the risk of mortality of severe morbidity if a serious diagnosis is overlooked. Sudden cardiac death syndrome was cited in this study as frequent worry hanging over doctors which may generate enormous anxiety. Parental anxiety and parent discomfort with uncertainty may certainly direct referral for specialist testing and consultation in such high-pressure consultations.

An important message from our study is that medical professionals can modify their own CWU. Although it remains contentious whether CWU is a trait or a learned phenomenon [5], our findings would point to the latter. Facing their fears and spending an elective or training period in a specialty which worries them was highlighted as an effective strategy to reduce CWU. Reaching out for support to colleagues both in paediatrics and in the specialty causing concern can build CWU. Learning to sit with the concern and ‘embrace the concern’ or discomfort with uncertainty was also highlighted as an important coping mechanism. In fact, this may be an integral component of the normal CWU journey for medical professionals dealing with patients. It is worth noting that much of the tactics our participants describe using to manage their CWU are not easily observable by trainees. They discuss internal reflection prompted by “gut” feelings. They describe contacting colleagues for sense-checks. These may go unnoticed by trainees and it is worth developing curricular activities to specifically share how they process CWU proactively. Previous work with medical students using reflective diaries has shown promise in this area [12] and could be adapted to supervisors and trainees sharing reflective diary accounts of CWU.

Our study also assessed how doctors cope with uncertainty in different working environments. Those in rural areas have less access to specialists and investigations and reported relying on CWU to a greater extent. The additional CWU challenges faced by clinicians in rural or sparsely resourced areas should be acknowledged in curriculum design. Digital enhanced learning facilities have proven effective in supporting trainees through challenges experienced in remote or rural practice [38,39]. Strategic mentorship could also potentially alleviate epistemic uncertainty by accessing expert knowledge [40]. Considering the availability and duration of rotations through a wide range of specialities needs to be examined in conjunction with service provision.

Furthermore, our data would support that medical professionals can learn to modify their patient/ care-givers CDR. Although professionals may have relatively high CWU, their ultimate actions are strongly influenced by moderators, in particular stimulus characteristics, (i.e. how the patient or, more commonly in paediatrics, care-giver, reacts to the child’s presentation and proposed management plan). Our participants identified that it was a challenge to separate their experience of uncertainty from that of the caregivers’. This is important as it subverts to some extent our thinking on how CWU teaching should occur. Uncertainty is the leading cause of psychosocial stress amongst patients [41]. If the physician’s CWU is actually only a secondary consideration, and the patient or caregiver’s CDR is ultimately a key factor in the management plan, we must move towards patient and public partnerships in providing appropriate CWU educational interventions. Recognition of the relational dynamics and preparing trainees to consider CWU in terms not just of ill-defined problems, but as patient-centred interactions is a key paradigm shift in considering programme design. Much of literature on training for clinical uncertainty focuses on supporting clinical reasoning [42,43]. Evidence indicates that parents feel more uncertainty in situations where they have less personal control over their child’s illness and when they did not have knowledge of the likely future healthcare journey for their child [44]. Participants alluded to these issues and their attempt to address them, but indirectly and indicated their eventual management strategies were a result of years of experience and some “trial and error”. Our work suggests that building educational activities that offer experience with clinically uncertainty underpinned by complex, socially influenced moderators are more likely to present educational opportunities that mirror the experiences our participants describe.

Our experienced clinicians provided a wealth of examples of how they attempt to increase caregiver CDR with the purpose of coming to a shared agreement on what they felt is clinically appropriate. They highlight ritualistic acts in putting patients at ease—performing a full examination, documenting vital signs, and records in front of the caregiver. They specifically highlighted the importance of investing in active listening and clearly communicating when the existence and extent of uncertainty, to build trust with the family. This approach has been shown to support relationship-building between physician and patients [45]. Our participants’ approach mirrors guidelines that routinely reference the need for open communication regarding uncertainty with patients [46]. Our participants indicate that this has come with experience, echoing the established reports that there is a paucity of CWU training in formal curricular activities [2], underlining the need to develop some. Adapting the framework for communicating uncertainty to postgraduate simulation has the potential to ensure trainees are equipped with a standardised patient-centred model for these challenging encounters [26].

The final message from our study is that doctors live in the real world, not an academic bubble. Strategies to build medical professional CWU or to reinforce patient/caregiver CDR are not immune from societal and parental pressures. Human factors, especially fear and anxiety, may override all logical explanations of uncertainty [47]. We live in a consumerist world, where some patients, despite efforts to allay their anxiety will either doctor-shop themselves or insist on referral for testing of benign conditions. Although cognitive and behavioral responses to uncertainty are important, perhaps mediating emotional responses remains the most challenging [15,48]. A mothers’ concern for her child may be driven by her intuition and “gut feelings”, which doctors should be very careful to dismiss. Although every case should be evaluated on a case-by-case basis, teaching strategies, where appropriate, to move caregivers from a situation of anxiety to calm may help alleviate their discomfort with uncertainty.

## Limitations

Ideally, we could have examined clinicians after they themselves conducted the clinical interaction with the child and parent. However, the COVID-19 pandemic prevented this possibility. Although validated by two doctors, the clinical scenario may emphasize some components of CWU but also deemphasize others. However, we believe this was a reliable lens through which to view this real-life clinical scenario. This study, although revealing several important findings, is limited to an Irish context and would have benefited from the involvement of doctors from several other countries. Although we studied general paediatricians and several doctors with different subspecialties we did not correlate CWU with specific speciality. Furthermore, we did not assess the impact of gender on CWU. The experiences of Irish physicians may be influenced by their training, which leans heavily on the importance of history and physical examination, limited access to cardiac imaging in some regional and rural hospitals, as well as the culture among some physicians of not referring unless there is a high index of suspicion of pathology. Conversely, medicolegal and parental anxiety are increasingly prevalent in Irish healthcare, which may also influence their comfort with uncertainty. Our results indicated that paediatricians felt inappropriate referral occurred when they were unable to manage caregiver CDR, highlighting the significant training approach needed to optimise clinician skills in supporting caregiver CDR.

## Conclusion

In conclusion, understanding how comfort with uncertainty works in the clinical arena is highly complex, context-specific and varies widely between different practitioners. Clinicians’ confidence in interpreting history and clinical findings and building a relationship with the patient/caregiver are key strategies in building CWU. Clinician CWU in the paediatric context is inextricably linked to caregiver CDR. Clinician CWU and caregiver CDR can be fostered and developed and is not a fixed trait. The complexities and central importance of social context in understanding CWU has important implications for how we develop educational activities to support clinician CWU and patient/caregiver CDR.

## Additional File

The additional file for this article can be found as follows:

10.5334/pme.1394.s1 Appendix. Interview Guide.
