# Supplementary material for: How Do Paediatricians Manage Comfort with Uncertainty in Clinical Decision-Making
Source: Perspect Med Educ. 2024 Oct 22;13(1):527–39. doi: 10.5334/pme.1394 (PMC11505027; doi:10.5334/pme.1394)
Supplement: Appendix. — Interview Guide. [file pme-13-1-1394-s1.pdf]

Appendix Interview Guide

What is your name?

How many years have you been practitioner as a consultant in paediatrics?

Do you work in a rural or urban setting?

Are you a general paediatrician or a subspecialist paediatrician?

What did you make of the clinical interaction between doctor, child and parent?

Were there any red flags you witnessed from the clinical interaction?

Where there any cues that either provided comfort or discomfort with uncertainty for you from the video?

What would your next course of action be in this clinical setting?

What factors build comfort with uncertainty for you dealing with patients?

What actions do you think build comfort with uncertainty for parents/caregivers?

Does greater experience affect your degree of comfort with uncertainty?

What factors damage or reduce your comfort with uncertainty?

How do you manage parental anxiety or pressure to refer even if you don't believe the child needs to be referred to a cardiologist?

Do you feel under pressure to refer patients to subspecialists even if you are comfortable with the diagnosis and plan of management?

Does modern society tolerate uncertainty?

Did COVID affect how you dealt with uncertainty in decision-making?
